# Epidemiology and the diagnostic challenge of extra-pulmonary tuberculosis in a teaching hospital in Ethiopia

**DOI:** 10.1371/journal.pone.0243945

**Published:** 2020-12-15

**Authors:** Balew Arega, Amdemeskel Mersha, Abraham Minda, Yitagesu Getachew, Alazar Sitotaw, Tefera Gebeyehu, Asnake Agunie

**Affiliations:** Yekatit 12 Hospital Medical College, Addis Ababa, Ethiopia; The University of Georgia, UNITED STATES

## Abstract

**Background:**

Ethiopia reported a high rate of extra-pulmonary tuberculosis (EPTB) and the cases are increasing since the last three decades. However, diagnostic evidence to initiate TB treatment among EPTB cases is not well known. Therefore, we described the epidemiology and assessed how EPTB is diagnosed in a teaching hospital in Ethiopia.

**Methods:**

We conducted a retrospective review among all adult EPTB cases diagnosed in Yekatit 12 Hospital Medical College from 2015 to 2019. Using a standardized data abstraction sheet, we collected data from patients’ medical records on sociodemographic, sites, and laboratory diagnosis of EPTB cases.

**Results:**

Of the 965 total TB cases, 49.8%(481) had a recorded diagnosis of EPTB during the study period. The mean age of EPTB patients was 32.9 years (SD±13.9) and 50.7% were males. Tubercular lymphadenitis (40.3%), abdominal (23.4%), and pleural TB(13.5%) were the most common sites of EPTB involvement, followed in descending order by the genitourinary, skeletal, central nervous system, abscess, breast, and laryngeal TB. We found a histopathology finding consistent with EPTB in 59.1% of cases, Acid-fast bacilli positive in 1.5%, and the rest diagnosed on radiological grounds. In the majority of cases, more than one diagnostic method was used to diagnose EPTB cases.

**Conclusions:**

Nearly half of TB patients had a recorded diagnosis of EPTB that comprise heterogeneous anatomical sites. All EPTB patients were started anti-TB therapy without definitive microbiology results. This indicates the diagnostic challenge of EPTB faced in our setting and proves to be significant for TB control in Ethiopia.

## Introduction

Pulmonary tuberculosis (PTB), which affects the lung, accounts for 85% of reported TB cases worldwide [1]. Currently, however, extra-pulmonary TB (EPTB) which involves the body other than the lung becomes a growing problem [2]. This change in epidemiology might be due to the emergence of the human immunodeficiency virus (HIV), the changing of TB control practices, increase in incidence of comorbid conditions, and the evolution of diagnostic tools [3]. The bacilli from the lung disseminate through the lymphatic or hematogenous system and subsequently affect single or multiple extra-pulmonary sites [4]. Lymph nodes, abdomen, pleura, bones, and meninges are the most common anatomic sites affected by EPTB, but the frequency varies with age, sex, and geographic area [5].

Since the EPTB affects virtually any organ and produces a wide spectrum of clinical manifestations, involves inaccessible sites, and the affected body fluids (mainly pleural and peritoneal) are paucibacillary in nature, it causes challenges in effective disease diagnosis and management [6]. These patients are commonly treated for other alternative diagnoses during their first visit to the health care facilities, which is mainly in primary health care settings. Even in the tertiary health care facilities, the majority have been started anti-TB therapy without bacteriology confirmed results because of the indolent diagnostic challenge [7]. These patients were delayed in diagnosis or misdiagnosed and underestimate the size of the problem at the community level.

Ethiopia is the 3^rd^ top country affected by EPTB globally and the case notification is almost equal to that of smear-positive PTB (SPPTB) and smear-negative PTB(SNPTB) [8]. The national TB programs, however, have focused on PTB because of transmissibility, and likewise, the majority of studies done in Ethiopia focused on PTB [9]. Some of the previously conducted studies in Ethiopia focused on the genotypic characterization of mycobacterium causing EPTB [8, 10–12] and other assessed diagnostic modalities accuracy and sensitivity for lymph nodes TB [13–29]. A detailed characterization incorporating sociodemographic, clinical, laboratory, and radiological features of adult EPTB cases is lacking in Ethiopia. Further, what proportion of the EPTB patients initiated anti-TB therapy based on confirmed bacteriology result is also not well known. Such information is important at the tertiary health care level because patients are transferred to the primary health care level to complete TB treatment and lack monitoring of treatment response. Thus, the aim of this study was twofold. First, we have described the epidemiology and sociodemographic characteristics of EPTB patients. Second, we evaluated the diagnostic and laboratory characteristics of EPTB cases who initiated anti-TB therapy at a teaching hospital in Ethiopia.

## Materials and methods

### Study setting

We conducted the study at Yekatit 12 Hospital Medical College (Y12HMC), which is a referral teaching hospital, Ethiopia. The hospital is situated in Addis Ababa city and has nearly 500 beds. It has treated approximately 310,000 patients from the city and its surroundings annually. There are different (more than 21) departments, wards, and clinics such as TB clinics in the hospital. The hospital received TB patients suspected of smear-negative TB, pediatrics TB, and EPTB referred from health facilities (health centers and primary hospitals) assigned under its coverage. Patients referred for other health problems might also have been diagnosed with TB. The presumptive diagnosis of EPTB in the hospital is made using clinical evidence of EPTB, acid-fast bacilli test (AFB), histopathology finding consistent with TB, and body fluid analysis of ETPB sites. Besides, X-ray, computed tomography (CT), magnetic resonance imaging (MRI), and ultrasound (US) were also used to diagnose TB. Mycobacterium culture has not been offered in the hospital. Despite this, Xpert MTB/RIF has been recently endorsed but it is not used consistently for diagnosis of adult EPTB cases because of primary indication for HIV/TB confection, presumptive multidrug-resistant TB, and pediatrics TB [30]. For this study, the laboratory diagnosis of EPTB at different anatomical sites was considered as follows:

- Lymph nodes TB (TBLN), genitourinary TB (GUTB), TB abscess, and laryngeal TB: histopathology of biopsy samples suggestive of TB taken from the respective anatomical site.
- Pleural, peritoneal, or TB meningitis: Exudative fluids (pleural, peritoneal, and cerebrospinal fluid (CSF)) with lymphocytic predominance were considered TB unless proven otherwise.
- Intestinal TB: CT description of thickened bowel loops was acceptable. The US description that suggests TB in other anatomical sites could also be considered as a diagnostic modality as much as it was used for the decision to initiate anti-TB therapy.
- Skeletal TB, central nervous system (CNS) tuberculoma, or spinal TB: MRI or CT evidence was considered sufficient evidence for initiating treatment.
- AFB test: a positive AFB test of any of the EPTB samples is highly suggestive of Mycobacterium species.
- Histopathology: typical histopathology finding for EPTB is the caseation granuloma of the biopsy sample. In our hospital, patients with a histopathology finding of non-caseation granuloma also start ant-TB therapy unless proven otherwise.

### Study design and population

We retrospectively enrolled all patients diagnosed with EPTB at the study area from 1^st^ January 2015 to December 30, 2019. All adult EPTB patients whose laboratory investigation result suggested EPTB and initiated TB treatment in the hospital were included. Pediatrics TB patients (<16 years old), PTB, and disseminated TB were excluded. Also, we excluded EPTB patients who were initiated anti-TB therapy only based on clinical diagnosis.

### Data collection tool and procedure

We retrieved EPTB patients’ medical charts from the main archive room using a medical record number (MRN) obtained from the hospital TB clinic. Then, we used a structured data abstraction sheet to collect data from the chart and TB registration logbook. The chart of the patients contains all the required data. We used a referral letter kept in the charts to get data about referral diagnosis. Three trained nurses collected the data and the principal investigators supervised the data collection. We extracted demographic data (age and sex), clinical presenting symptoms, duration of symptoms, EPTB types, previous TB, complete blood count (CBC) results, liver enzymes tests results, erythrocyte sedimentation rate (ESR) result, EPTB investigations (body fluid analysis, AFB, imaging, and histopathology). EPTB types were registered as TBLN (extrathoracic), abdominal TB (ATB), pleural TB, CNS TB (brain and spine), intestinal TB, skeletal TB(bone TB, joint TB), genitourinary TB (GUTB) (testes, epididymis, ovary, endometrium, fallopian tube, cervix, vagina and kidney), breast TB, laryngeal TB, pericardial TB, and other rare localization. We counted the total number of TB patients registered to determine the proportion of the different forms of TB (SPPTB, SNPTB, or EPTB).

### Ethical considerations

We obtained ethical approval from the Y12HMC Ethical Committee. The institutional review board waived the need for written informed consent from participants as the study collects only secondary data. To keep the privacy of the patients, we omitted the name of patents during data collection and excluded the chart number on publication. Besides, we ensured the confidentiality of data by keeping the collected data in a locked secure cabinet and the electronic database secured in a password-protected computer.

### Data analysis and statistics

We carried out the data entry using Epinfo version-7 and statistical analyses by software package SPSS version-20 (IBM Corporation, NY, and the USA). The descriptive statistical analysis included median and range for continuous variables, frequencies, and proportions for categorical variables. We presented data using tables and figures.

## Results

### Sociodemographic and clinical characteristics

**Fig 1:**
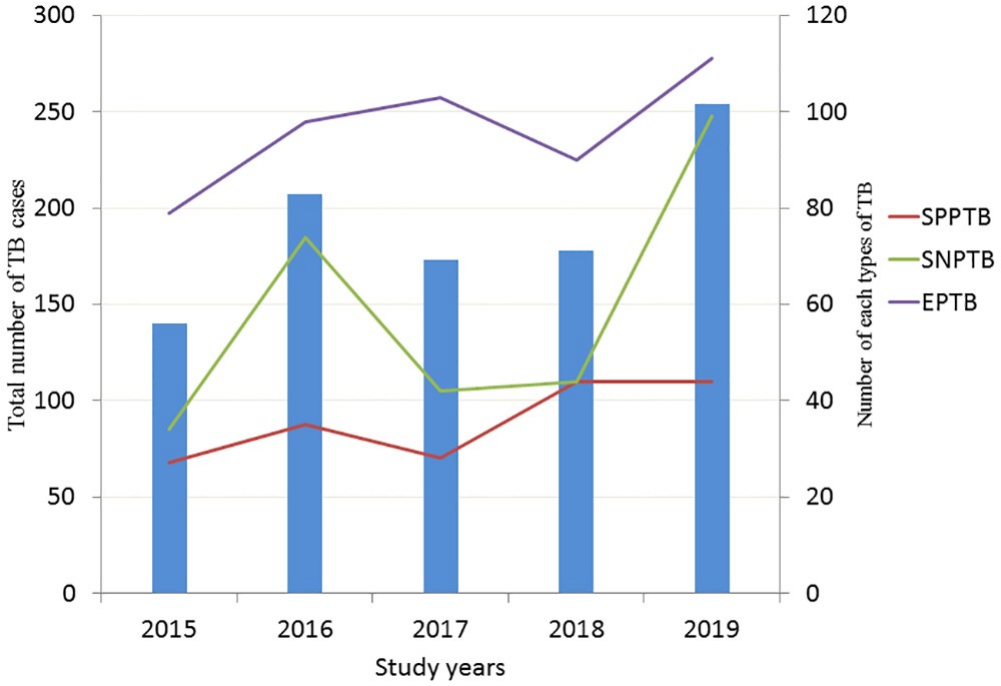
The proportion of EPTB at teaching hospital in Addis Ababa, Ethiopia, 2015 to 2019.

During the period under review (2015 to 2019), a total of 965 TB patients were registered for treatment. Among these,50.2%(484) were PTB and 49.8%(481) were EPTB patients. The proportion of EPTB cases ranged from 47.3% to 56.4% in the study period (Fig 1).

**Fig 2:**
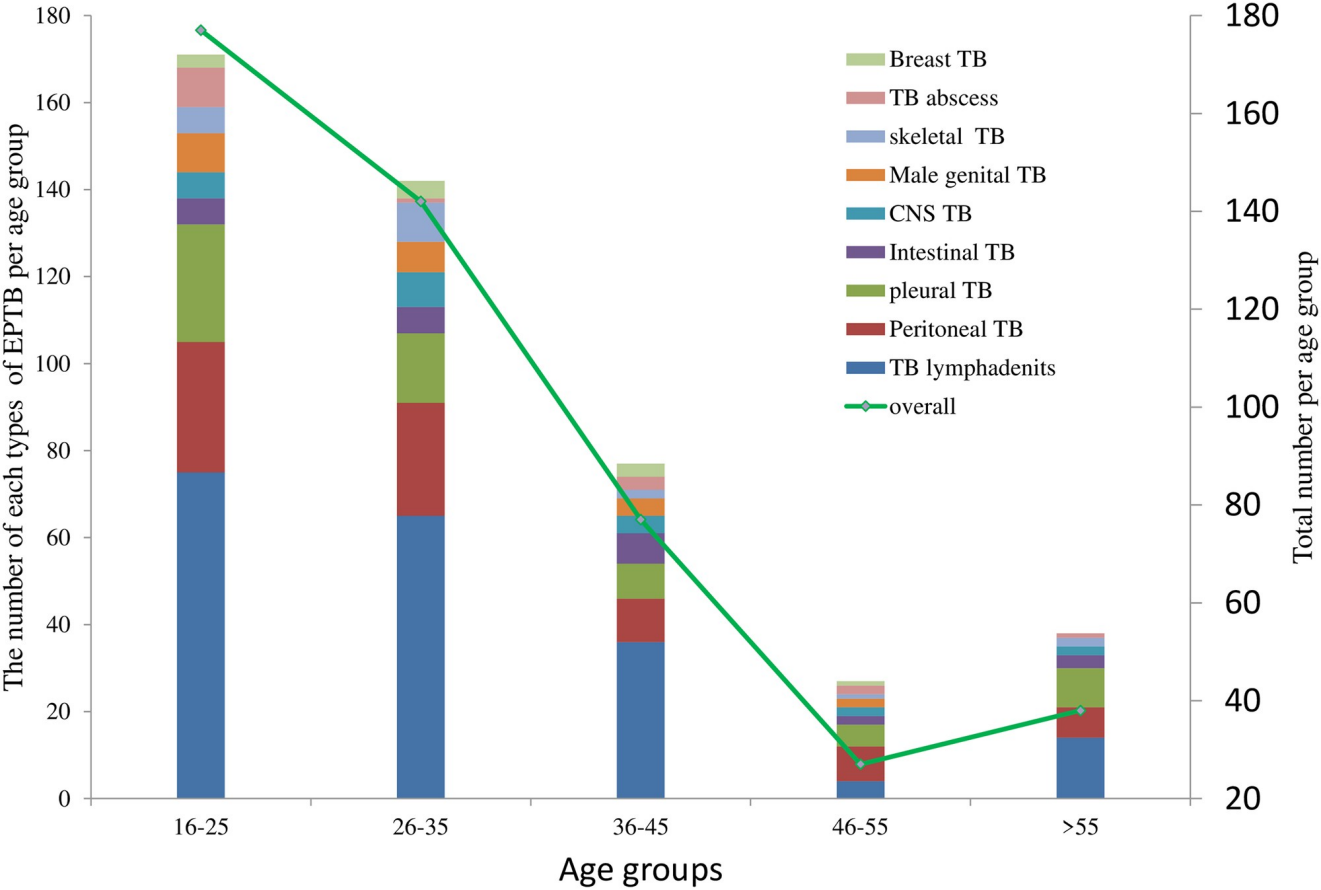
Age group distribution of EPTB at teaching hospital in Addis Ababa, Ethiopia, 2015 to 2019.

The mean age of EPTB patients was 32.9 years (SD±13.9) and 50.7% (244) were male. The majority,36.8%(177) of the EPTB cases were in the age range 16–25 years followed by age range 26–35 years, 31.2%(150). As presented in Fig 2, the proportion of EPTB generally decreased with aging.

Based on HIV screening, 54.2% (261) were tested for HIV and the rest 46.8% (220) did not know their HIV status (unknown). Among HIV tested, 24.9% (65) were HIV positive, of which 35.4% (23) knew their HIV positive result for the first time (new cases). Previous TB treatment, Diabetes mellitus, chronic liver diseases, and cardiac diseases were documented in 1.24% (6, each) and 0.6% (3, each), respectively. The majority, 91.5% (440) of the EPTB patients were referred from other health facilities, of which 27.5% (121) suggested TB in the referral letters (Table 1). The mean duration of symptoms (signs) before they visited (referred) to the study area was more than three months. We found that patients initiated anti-TB therapy within two weeks of visiting the study area (health setting delay).

**Table 1:** Socio-demographic and clinical characteristics of EPTB patients at a teaching hospital in Addis Ababa, Ethiopia, 2015 to 2019.

Sex	Frequency (%)
HIV status	
Positive	65(13.5)
Negative	196(40.7)
Unknown	220 (45.7)
Documented TB constitutional symptoms	
Night sweating	119(24.7)
Low-grade fever	113(23.5)
Loss of appetite	133(27.7)
Weight loss	121(25.2)
Easily fatigability	88 (18.3)
Referral	
Governmental health facilities	112(23.3)
Private health facilities	328(68.2)
Self-referral	41(8.5)
Referral diagnosis(n = 440)	
Suggested TB	121(34.3)
Not suggested TB	289 (65.7)
Duration of symptoms	14.9 ± 21.9 weeks
Health setting delay	12.5 ±22.6 days

*The time from a visit of the study area to initiation of ant-TB therapy.

### Radiological and hematologic findings of EPTB cases

We found a documented chest-ray report in 31.6% (152) of the EPTB cases. The result showed a normal x-ray report in 44.1% (67), pleural effusion in 42.8% (65), and pneumonia in 7.2% (11). The rest 6.6% (10) had other chest x-ray findings (old fibrosis, cardiomegaly).

Complete blood cell count (CBC) was found documented in 380 of the EPTB cases. Among these, normal white blood cells (WBC) and platelet count were found in 80% (304) and 78.4% (299) of patients, respectively. As presented in Table 2, an equal proportion of females and males had low hemoglobin levels, 41.7% (100). The erythrocyte sedimentation rate (ESR) was high in more than three–fourth of male and female EPTB patients with documented ESR result (n = 242). The value of aspartate aminotransferases (AST) and alanine aminotransferase (ALT) was normal in 77.13% (253) and 80.3% (253) of EPTB patients with a documented liver enzyme analysis, respectively.

**Table 2:** Hematologic and liver enzyme among EPTB patients at a teaching hospital in Addis Ababa, Ethiopia, 2015 to 2019.

Variables	Frequency (%)
**White blood cell (n = 380)**	
Leukopenia (<3.99x10^3^)	30(7.9)
Normal (4–11*10^3^)	304(80)
Leukocytosis (>11x10^3^)	46 (12.1)
Neutrophil ((Mean±SD %)	63.9±12.2
Lymphocyte (Mean±SD %)	25.4±12.0
**Platelet (/μl)(n = 380)**	
Low (<150x10^3^)	32(8.42)
Normal (150-450x10^3^)	298(78.42)
High (>451x10^3^)	50(13.16)
**Hemoglobin(g/dl)(n = 380)**	
Female (n = 187)	
Low (<12g/dl)	78 (41.7)
Normal (12-15g/dl)	96(53.3)
High (>15g/dl)	13(6.9)
Male (n = 194)	
Low (<13g/dl)	81(41.7)
Normal (13-17/dl)	105(54.1)
High (>17)	8(4.1)
**Erythrocyte sedimentation rate(ESR) (n = 242)**	
Male (n = 124)	
Normal (≤15mm/hr)	27(21.8)
High (>15mm/hr)	97(78.2)
Female (n = 118)	
Normal (≤20mm/hr)	26(22)
High (>20mm/hr)	92(78)
**Liver enzymes**	
Aspartate aminotransferases (AST)(n = 328)	
<35 IU/L (normal)	253(77.13)
35–70 IU/L	54(16.46)
>71 IU/L	21(6.4)
Alanine aminotransferases (ALT)(n = 315)	
≤35 IU/L(normal)	253(80.32)
35–70 IU/L	42(13.33)
≥71 IU/L	20(6.35)
Alkaline phosphatase(ALP)(n = 298)	
≤150 IU/L (normal)	68(22.82)
≥150–300 IU/L	161(54.03)
≥301 IU/L	69(23.15)

### Clinical presentation and diagnosis of EPTB cases

**Fig 3:**
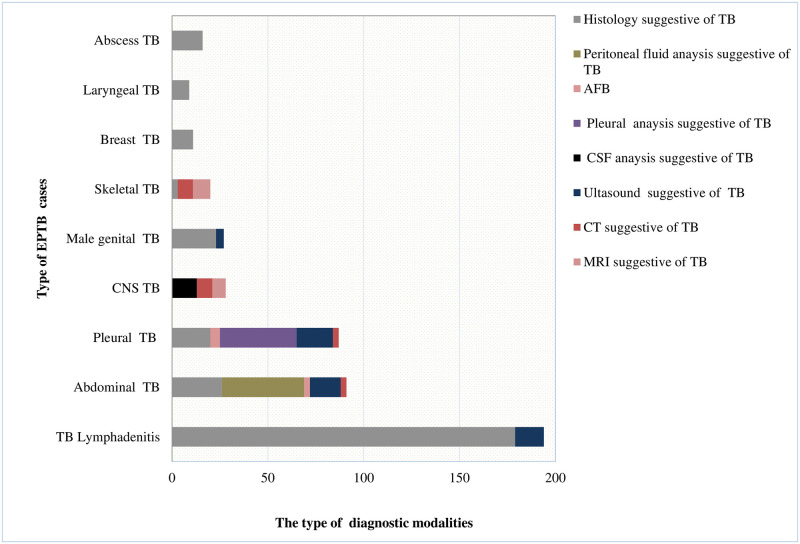
Diagnosis of EPTB at teaching hospital in Addis Ababa, Ethiopia, 2015 to 2019.

Fig 3 presents the diagnostic characteristics of the EPTB cases studied. More than half of EPTB cases, 59.0% (284), were diagnosed with histopathology findings consistent with TB. Only 1.5% (7) had a documented AFB positive results, of which three also had a histopathology result consistent with TB.

**Fig 4:**
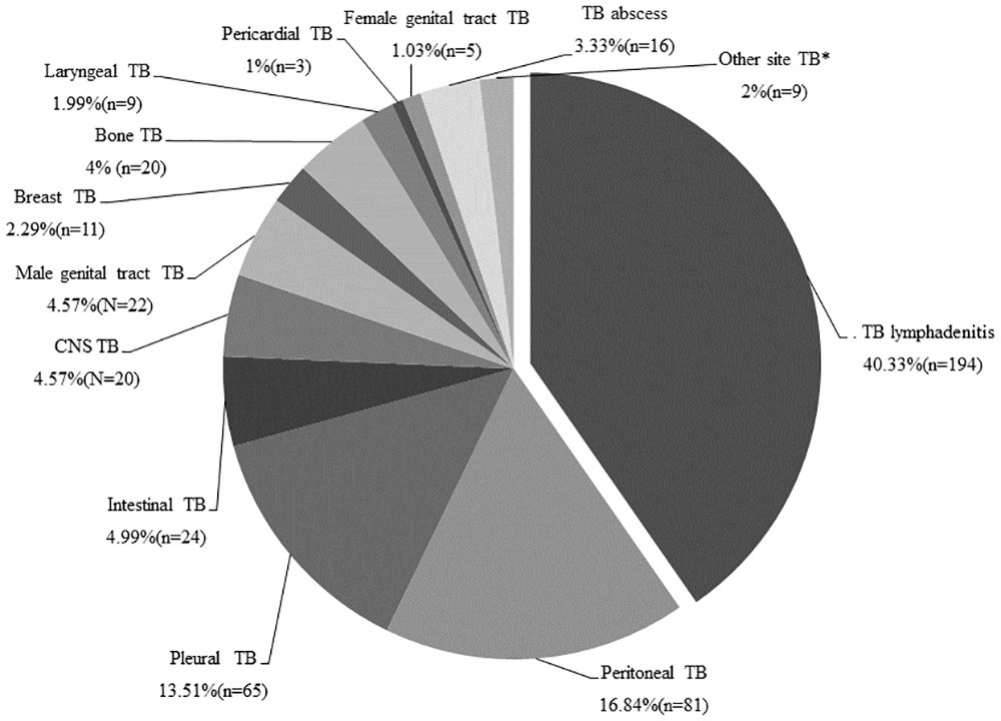
Anatomical sites of EPTB in Addis Ababa, Ethiopia, 2015 to 2019.

The most frequent sites of extra-pulmonary involvement were lymph nodes, 40.3% (194) (Fig 4). Most, 63.9% (124) occurred among females. The swelling of lymph nodes was documented in 87.7% (170) of these patients. The diagnosis in the majority, 92.3% (179) of these cases was made with histopathology finding suggestive of TB.

The second most common EPTB site was ATB (peritoneum, liver, spleen, and intestine), 23.4% (111). The majority 72.9% (81) had peritonitis and the remaining 27.1% (30) had no peritonitis. A slightly higher proportion, 51.4% (57) of ATB occurred among females. The documented presenting symptoms included weight loss, 50.5% (56), loss of appetite,43.2%, (48), fever, 37.8% (42), abdominal pain,36.9% (44), night sweating, 32.4% (36), and abdominal distension,18.9% (21). Among ATB with peritonitis,62 had a documented fluid analysis result. The mean lymphocyte percentage and mean protein value was 71.1% (SD±16.9%) and 5.4g/dl (SD±1.8g/dl), respectively (Table 3). Twenty-two (35.5%) had the histopathology findings suggestive of TB Two (7.4%) patients had AFB positive results among the 27 patients who had documented ascitic fluid analysis.

**Table 3:** Body fluid analysis result at a teaching hospital in Addis Ababa, Ethiopia, 2015 to 2019.

Variables	Frequency
**Peritoneal fluid analysis (n = 62)**	
WBC (mean + SD cell/l)	1438.4±1182.9
Neutrophil (mean + SD %)	28.7±16.8
Lymphocyte (mean + SD %)	71.1±16.9
Protein (mean + SD g/l)	5.4±1.8
Glucose (mean + SD gm/l)	87.9 ±48.8
**Pleural fluid analysis (n = 64)**	
WBC (mean ± SD cell/l)	2249.4 ±2110.
Neutrophil (mean ±SD %)	23.8+13.7
Lymphocyte (mean ±SD %)	76.1±13.7
Protein((mean + SD gm/l)	4.8±1.12
Glucose (mean + SDgm/dl)	50.8±26.4
**Cerebrospinal fluid (n = 13)**	
WBC (mean + SDcell/μl)	786.8±924.2
Neutrophil (mean + SD %)	19.5±8.9
Lymphocyte (mean + SD %)	79.7±9.2
Protein (mean ± SDgm/l)	7.8±0.9
Glucose (mean + SDgm/l)	50.7±26.4

Pleural TB was the third most frequent involvement of EPTB site, 13.5% (65). Most, 77.8% (51) occurred among males. The documented presenting symptoms were weight loss, 43.1% (28), loss of appetite, 49.2% (32), fever, 52.3% (34), night sweating, 47.7% (31), cough, 41.5% (27), and chest pain, 12.3% (8). The diagnosis of all these patients relied on pleural fluid analysis. The mean percentage of lymphocyte was 76.1% (SD±13.7%) and the mean protein value was 4.8g/dl (SD±1.12g/dl). Twenty (30.8%) patients had histopathology findings suggestive of TB (Table 3). AFB test was documented among 31 patients and five (7.8%) had AFB positive results.

Genitourinary TB (GUTB) was diagnosed among 5.6% (27) patients during the study period. Of these, the majority, 81.5%, (22) were male GUTB ((testes (10), scrotal (7), epididymis (5)) and the remaining, 18.5%, (5) were female GUTB ((cervix (2), endometrial (2) and ovarian (1)). The documented presenting symptoms were swelling of the affected area, 66.8% (18), night sweating 33.3% (9), loss of appetite, 29.6% (8), weight loss, 25.9% (7), fever, 22.2% (6), and abdominal pain,11.1% (3). All male and three female GUTB had histopathology findings suggestive of TB.

Twenty (4.2%) patients were diagnosed with CNS-TB during the study period. Thirteen cases (65%) had TB meningitis (TBM) and the rest seven (35%) had TB tuberculoma. The documented presenting symptoms were headache, 55% (11), loss of appetite, 45% (9), weight loss, 45% (9), fever, 40% (8), and vomiting, 15% (3). We found a 79.7% (SD±9.2%) mean percentage of lymphocyte and 7.8 gm/dl (SD±0.9g/l) mean protein value of CSF analysis (Table 3). No AFB positive result was found among thirteen CSF analyzed.

Skeletal TB was diagnosed among 4.2% (20) (spine (16), hip (2), elbow (1), and knee arthritis (1)) patients. Sixteen (80%) of these cases were spinal TB. The documented presenting symptoms and/signs were back pain 65% (13), night sweating, 35% (7), lower extremity pain and weakness, 15% (3), fever 25% (5), and swelling of the affected joint, 20% (4). Two hip joint TB patients were diagnosed with MRI. The MRI showed adjacent soft tissue hyperintensity and minimal effusion. The knee and elbow joint TB arthritis patients had a histopathology exam revealed granulomatous inflammation with caseous necrosis. An equal number (8 each) of spinal TB patients were diagnosed using the CT scan and MRI as revealed in Fig 3.

We found 3.3% (16) TB abscess cases. The majority, 62.5% (10) of them occurred among males, and 37.5% (6) patients had psoas abscess. The documented presenting symptoms and/signs were swelling of the affected area, 56.3% (9), fever, 50% (8), loss of appetite, 37.5% (6), right lower/left quadrant pain, 37.5% (6), and night sweating, 18.8% (3). All patients with TB abscess had histopathology findings suggestive of TB.

During the study periods, 2.5% (9) patients presented with laryngeal TB. All patients had a change of voice (hoarseness) at presentation and evaluated at the eye, neck, and throat (ENT) clinic. We found that histopathology findings suggest TB in all of these patients.

Three patients (2.5%) presented with pericardial TB. All of these were found to have pericardial effusion in the echocardiographic study. The other three patients diagnosed with EPTB involved two organs in different cavities (pericardial and liver, pleural and liver, peritoneum, and pleural, one each). Lastly, one case of skin TB was diagnosed in a 21-year-old male who presented with a one-year duration of ulcer and histopathology finding suggested TB.

## Discussion

In this study, we set out to explore the epidemiology and diagnostic challenge of EPTB in teaching hospitals in Ethiopia. We found that close to half of the total TB patients in the five years had a recorded diagnosis of EPTB. Different types of EPTB occurred at a different frequency, with TBLN and ATB being the most commonly seen types. Consistent with our initial anticipation, only a few (1.5%) of EPTB patients had documented AFB positive results and the rest diagnosed with either histopathology or imaging.

Globally, the proportion of EPTB has been increased in the past decades yet remarkable differences have been shown across countries [31–33]. In the same way, the rate showed a steady increase since the 1990s in Ethiopia [8]. In this study, we found that nearly half of the total TB cases were EPTB and the proportion was stable between 2015 and 2019. The report from the Ethiopian ministry of health indicated that EPTB ranged from 20% to 45% along a south to north geographic axis and it accounted for roughly one-third of the new cases of TB in the country [12, 34]. The sub-notifications of EPTB due to the difficulties in EPTB diagnosis and the lack of access to adequate diagnostic infrastructure might underestimate the observed EPTB in the country. In this study, however, the magnitude of EPTB might be overestimated for the following reason. First, we conducted the study among referred patients for TB or other illnesses to a tertiary hospital. Second, because the diagnosis in the majority was not microbiologically confirmed, other mimic cases might be considered as EPTB. Consequently, all EPTB patients who started anti-TB therapy might not be EPTB cases. Generally, the rise in the incidence of EPTB in Ethiopia needs specific programs and resources to improve the diagnosis in the country.

We found that EPTB patients in this study were relatively young (mean age 32 years) and the proportion decline with aging. Likewise, a study in Saudi Arabia presented a high prevalence of EPTB among productive age groups [35]. Most TB cases in the young population might indicate the ongoing TB transmission in the community. The decline in the proportion of EPTB among the elderly might be related to the functional decline of monocytes and macrophages, the principal mediator of TB pathogenesis, during aging. They express a low level of Toll-like receptors from activated splenic and peritoneal macrophages and an altered secretion of several chemokine and cytokine [36, 37]. This might decrease the sensitivity of histopathology diagnosis because of the low granuloma formation in the aged population.

Although duration of symptoms differs based on the types of EPTB cases, the overall mean symptoms duration was three months in this study. In contrast, our country’s national guideline considered patients delay when patients do not seek health care for more than three weeks from date of onset for the illness [38]. However, the delay in our study might be resulting from the following reasons. First, due to the indolent nature of EPTB, the symptoms were not easily recognized by patients like the PTB. In our country, a cough of more than two weeks’ duration was well-publicized in the multimedia. Second, as this study was done in a teaching hospital, the delay might be a referral delay (primary health care to the hospital) or the patient’s delay. Lastly, it could also represent a failure of health providers to recognize EPTB cases. Generally; our country’s primary health care providers should consider EPTB as a diagnostic possibility and familiar with the common clinical symptoms and signs to prevent mortality due to unnecessary delay.

HIV facilitates the dissemination of TB from the lung to other sites. This is mainly due to low or no granuloma formation and functional disruption of the local immune response within the granuloma [39]. For example, EPTB was reported in 10–34% of non-HIV cases, and 50–70% of patients co-infected with HIV [40, 41]. Besides, a previous study in our country [42] reported that almost half of EPTB patients were diagnosed with HIV. On the contrary, in this study, 45.7% of EPTB cases had unknown HIV status (not documented or tested). In the same way, a recent meta-analysis revealed that about one-third of TB patients did not know their HIV status [43]. In the last one and half decades, Addis Ababa reported the highest prevalence rate of HIV infection in the country. So, this finding indicates a higher number of HIV-positive patients could be missed in this city [44].

In keeping with epidemiological data from other settings [45–47], we found that TBLN followed by abdominal TB were the commonest EPTB sites. Moreover, it was worth noting that in most of the EPTB studies conducted in Ethiopia so far, TBLN was found to be one of the most frequently observed types of EPTB [10, 12, 15, and 17]. In contrast, many other reports have shown pleural effusion as the most frequent form of EPTB [48, 49] which is the third common EPTB site in this study. The other EPTB sites such as urogenital, CNS, bone/joint, breast and the pericardium were also reported with considerable proportion. The inclusion criteria, the prevalence of HIV, and the diagnostic modalities can be the reasons for variations of different forms of EPTB across counties. In Ethiopia, the importance of all forms of EPTB has not yet been ascertained due to a lack of diagnostic facilities, difficulties in diagnosis, and lack of the national program focus.

Histopathology examination of affected sites and tissues is usually recommended for the diagnosis of EPTB patients in our study hospital. Although, the typical histopathological finding for EPTB is a caseation granuloma [7] patients with non-caseation granuloma can also initiate TB treatment in our setting. This decision is made considering the high prevalence of TB in the country and the lack of or non-availability of definitive diagnostics tools such as the mycobacterial culture technique. In this study, histopathology was positive in 59.0% of cases. This is equivalent to study in the northeast (62.3%) [39] of the country but slightly higher than a study in Sudan (45.5%) [48], Saudi Arabia (42.9%) [50], and in northern Ethiopia (39.7%) [51]. In contrast, 80% of patients with EPTB had histopathology positive results in Hong Kong [52].

In our setting, patients with Lymphocyte-predominant exudates (pleural, peritoneal, and CSF) with high protein and low or normal glucose levels have been initiated anti-TB therapy. In this study, the majority of TB peritonitis and pleural TB patients started TB treatment based on the lymphocyte-predominant fluid analysis result. In accordance, a lymphocyte-predominant exudates [53–55] fluid of the pleura or peritoneum were also considered for the decision to start TB treatment in other developing countries. Since other illnesses especially intestinal carcinomatosis that revealed lymphocyte-predominant fluid analysis might mislead the diagnosis. So, patients who have initiated TB treatment based on this criteria need strict monitoring of the treatment response and early decision.

Mycobacteria culture is the gold standard for definitive diagnostic [7] but it has been used mainly for research purposes and avail in drug resistance TB treatment center in our country. Consistent with studies in other developing countries (0.5%-19.20%) [48, 50, 56], we found that only 1.5% had AFB positive results. In contrast, EPTB cases were bacteriology confirmed in 58–76% [57–59] in developed countries. The lack of verification of EPTB cases with culture and molecular techniques might lead to drug-resistant TB, diagnostic delays, misdiagnoses, and increased mortality rates. This mandates the need to: i) develop and adopt a standardized diagnostic scoring system in our hospital and ii) monitor the treatment response at the diagnostic sites, rather than referring back to the primary health care provider with limited education, to identify early treatment failure [29].

The study has the following limitation. First, it is a hospital-based retrospective study and hence findings cannot be generalized to the community at large. Second, we were not able to get additional information not included in the patient’s chart or registration book. Third, since the patients were referred to the nearby primary health care system to complete TB after diagnosis, we could not get information about the treatment response. Following the patient’s response to treatment would have been useful for clinicians given the diagnostic limitations.

In conclusion, the study provides an insight into the epidemiology and diagnostics profile of EPTB in Ethiopia. Our data shows that the EPTB was the common disease entity and comprises a heterogeneous anatomical site. So, the study illustrates the varied presentations of EPTB that should be known by healthcare workers throughout the country. We also observed that majority of EPTB cases were diagnosed. This predisposes to a high likelihood of misdiagnosis, due to lack of bacteriology confirmed result and monitoring treatment response, of PTB cases. Therefore, research needs to be conducted to better define clinical predictors and microbiological tests to improve early detection. Besides, a prospective study is needed to determine the treatment response of EPTB cases that started anti-TB therapy based on the presumptive diagnosis, important to explore misdiagnosis.

## Abbreviations

ATB: Abdominal tuberculosis
EPTB: Extra-pulmonary tuberculosis
GUTB: Genitourinary tuberculosis
HIV: Human immunodeficiency virus
TBLN: Lymph node tuberculosis
